# 胸部CT二维与三维特征对肺磨玻璃结节浸润性的诊断效能对比

**DOI:** 10.3779/j.issn.1009-3419.2022.102.39

**Published:** 2022-10-20

**Authors:** 鸿亚 王, 赫 杨, 子成 刘, 亮 陈, 心峰 徐, 全 朱

**Affiliations:** 210029 南京，南京医科大学第一附属医院/江苏省人民医院胸外科 Department of Thoracic Surgery, Jiangsu Province Hospital, The First Affiliated Hospital of Nanjing Medical University, Nanjing 210029, China

**Keywords:** 肺肿瘤, 放射学, 计算机断层扫描, 计算机三维成像, 体积, Lung neoplasms, Radiology, Computed tomography, Computer-generated 3D imaging, Volume

## Abstract

**背景与目的:**

目前越来越多的研究通过肺结节三维特征预测浸润性腺癌(invasive adenocarcinoma, IAC)，但少有研究证实与传统的肺结节二维特征相比，三维特征诊断IAC更有优势。本文分别从三维与二维层面分析IAC与非浸润性腺癌组胸部计算机断层扫描(computed tomography, CT)特征差异，比较二者鉴别IAC的优劣，其中非浸润性腺癌组包括前驱性腺体病变(precursor glandular lesions, PGL)及微浸润性腺癌(minimally invasive adenocarcinoma, MIA)。

**方法:**

收集2019年1月-2019年12月1, 045例肺磨玻璃结节(ground-glass opacity, GGO)手术患者临床资料，回顾性分析术前CT影像特征与病理学结果的相关性，由多因素*Logistic*回归分别按二维与三维分类筛选出鉴别IAC的独立影响因素，通过受试者工作特征(receiver operating characteristic, ROC)曲线找出鉴别IAC的cut-off值，并以约登指数评估诊断IAC的能力。

**结果:**

二维因素中结节最大径、实性成分最大径、纵隔窗结节最大径以及三维因素中结节总体积、实性部分体积、平均CT值均是诊断IAC的独立危险因素。将各项指标通过约登指数排列：实性部分体积(0.601) > 结节体积(0.536) > 实性成分最大径(0.525) > 结节最大径(0.518) > 纵隔窗结节最大径(0.488) > 实性成分体积占比(0.471) > 肿瘤消失率(tumor disappearance ratio, TDR)(0.468) > 实性成分占比(consolidation/tumor ratio, CTR)(0.394) > 平均CT值(0.380)。

**结论:**

三维层面CT特征诊断IAC优于二维层面，实性成分大小优于结节总体大小。

## Abstract (en)

**Background and objective:**

At present, more and more studies predict invasive adenocarcinoma (IAC) through three-dimensional features of pulmonary nodules, but few studies have confirmed that three-dimensional features have more advantages in diagnosing IAC than traditional two-dimensional features of pulmonary nodules. This study analyzed the differences of chest computed tomography (CT) features between IAC and minimally invasive adenocarcinoma (MIA) from three-dimensional and two-dimensional levels, and compared the ability of diagnosing IAC. The non-invasive adenocarcinoma group includes precursor glandular lesions (PGL) and minimally invasive adenocarcinoma (MIA).

**Methods:**

The clinical data of 1, 045 patients with ground glass opacity (GGO) from January to December 2019 were collected. Then the correlation between preoperative CT image characteristics and pathological results were analyzed retrospectively. The independent influencing factors for the identification of IAC were screened out according to two-dimensional and three-dimensional classification by multivariate *Logistic* regression and the cut-off point for the identification of IAC was found out through the receiver operating characteristic (ROC) curve. At last, the ability of diagnosing IAC was evaluated by Yoden index.

**Results:**

The diameter of nodule, the diameter of solid component, the diameter of mediastinal window nodule in two-dimensional factors, and the volume of nodule, the volume of solid part and the average CT value in three-dimensional factors were independent risk factors for the diagnosis of IAC. These factors were arranged by Yoden index: solid partial volume (0.601) > nodule volume (0.536) > solid component diameter (0.525) > nodule diameter (0.518) > mediastinal window nodule diameter (0.488) > proportion of solid component volume (0.471) > 1-tumor disappearance ratio (TDR) (0.468) > consolidation tumor ratio (CTR) (0.394) > average CT value (0.380).

**Conclusion:**

The CT features of three-dimensional are better than two-dimensional in the diagnosis of IAC, and the size of solid components is better than the overall size of nodules.

目前肺癌是全球发病率第二、死亡率第一的肿瘤^[1, 2]^，在国内，肺癌发病率逐年上升^[3]^。随着人们健康意识的提高及薄层胸部计算机断层扫描(computed tomography, CT)的普及，越来越多的以磨玻璃结节为主要表现的早期非小细胞肺癌被发现及被干预。世界卫生组织(World Health Organization, WHO)2021年肺肿瘤分类将非典型腺瘤样增生(atypical adenomatous hyperplasia, AAH)和原位腺癌(adenocarcinoma *in situ*, AIS)单独归为前驱性腺体病变(precursor glandular lesion, PGL)，微浸润性腺癌(minimally invasive adenocarcinoma, MIA)与浸润性腺癌(invasive adenocarcinoma cancer, IAC)属于浸润性病变^[4, 5]^。研究^[6-8]^表明，PGL与MIA患者术后5年生存率达到或接近100%，IAC则预后相对较差。不同时期的肺腺癌手术治疗措施也不相同^[9]^，例如PGL与MIA通过肺楔形切除术或肺段切除术即可达到很好的治愈效果，而IAC更适合选择肺叶切除术^[7]^。因此，术前通过胸部CT预测肺结节的病理类型以指导治疗方法的选择是很重要的。

目前我们通过胸部CT预测病理结果主要依靠肺窗结节最大径、结节实性成分最大径、纵隔窗结节最大径、实性成分占比(consolidation tumor ratio, CTR)、肿瘤消失率(tumor disappearance ratio, TDR)以及形态学特征如分叶征、毛刺征、支气管充气征、空泡征、胸膜牵拉等二维层面CT特征^[10-12]^。实性成分的存在被认为对肺腺癌的浸润性具有重要预测价值，如日本JCOG系列研究根据CTR的大小为≤2 cm的肺结节制定合适的手术方式，这在一定程度上肯定了二维特征对肺结节良恶性诊断价值。随着CT读片软件的完善与发展，我们可以在三维层面上测量肺结节的总体积、实性成分体积以及实性成分体积占比、肺结节平均CT值，根据研究，它们对肺结节病理同样有良好的预测效果^[13-15]^。然而，目前对比二维及三维特征对诊断肺结节浸润性诊断价值的研究较少，本文通过采用受试者工作特征(receiver operating characteristic, ROC)曲线比较二者对肺浸润性腺癌的诊断的能力，希望在临床工作中对诊断肺腺癌使用指标的选择提供帮助。

## 1 材料与方法

### 1.1 临床资料

(1) 纳入标准：①患者术后病理提示为AAH、AIS、MIA、IAC; ②患者术前未行新辅助治疗; ③患者手术肺叶肺结节单发，多发肺结节时仅研究浸润程度最高者。(2)排除标准：①患者合并有其他影响测量准确性的肺部疾病，例如：肺炎、肺间质性疾病、肺结核等; ②病灶最大径 > 3 cm; ③肺结节为纯实性(我们将肺结节实性成分体积占比大于95%视作纯实性); ④既往患侧肺手术史; ⑤胸部CT模糊; ⑥患者病历资料或影像学资料保存不完整。本研究回顾性分析2019年1月-2019年12月在南京医科大学第一附属医院胸外科接受手术治疗的肺腺癌患者1, 045例，排除影像学资料丢失84例，合并肺部炎症10例，纤维增生1例，神经内分泌肿瘤2例，转移癌7例，同侧肺术后25例，CT成像模糊患者1例，结节最大径 > 3 cm患者94例，纯实性结节35例，剩余786例患者纳入研究。其中男性患者78例，女性患者708例，年龄23岁-86岁，平均年龄(55.15±11.62)岁。PGL患者95例(AAH 15例，AIS 80例)，MIA患者221例，IAC患者470例。

### 1.2 影像学方法

#### 1.2.1 图像采集

采用Siemens Sensation 64层螺旋CT机，MEDRAD STELLANT高压注射器。扫描参数：管电压120 kV，采用自动管电流调制技术，准直0.625 mm，扫描范围：肺尖至膈顶部。采用1.0 mm层厚、0.5 mm层距对扫描数据进行重建后传至工作站进行后处理。

#### 1.2.2 图像处理

二维图像处理：调整肺窗窗宽为1, 600 Hu，窗位为-600 Hu; 纵隔窗窗宽为400 Hu，窗位为40 Hu。由两名从事胸部读片的放射科医生与一名胸外科医生共同读片，并在读片前对数据进行匿名处理。其中肺窗结节最大径、结节实性成分最大径、纵隔窗结节最大径由三名医生分别测量后取平均值。CTR=肺窗实性成分最大径/肺窗结节最大径，TDR=1-纵隔窗结节最大径/肺窗结节最大径(图 1，图 2)。

**图 1:**
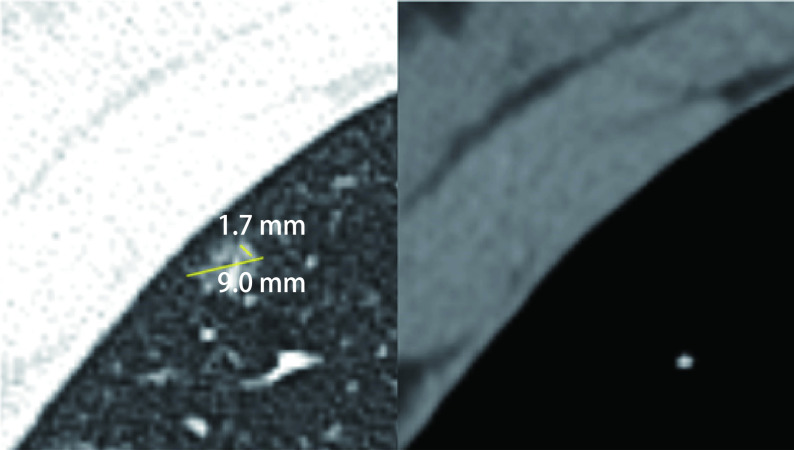
MIA患者CT表现。患者，女，43岁。结节最大径9.0 mm，实性成分最大径1.7 mm，CTR 18.89%，纵隔窗最大径0，TDR 0。术后病理为MIA。 CT of patients with MIA. The patient was a 43-year-old woman. The diameter of the nodule was 9.0 mm. The diameter of the solid component was 1.7 mm. CTR was 18.89%. The diameter of mediastinal window nodule was 0. TDR was 0. The postoperative pathology was MIA. CT: computed tomography; MIA: minimally invasive adenocarcinoma; CTR: consolidation tumor ratio; TDR: tumor disappearance ratio.

**图 2:**
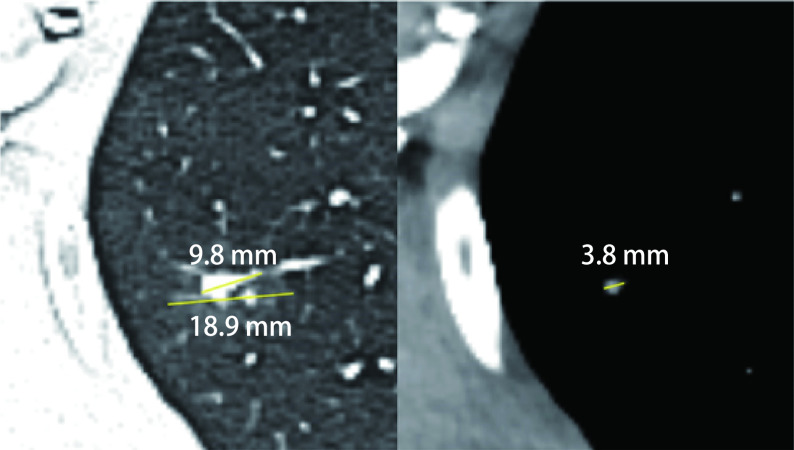
IAC患者CT表现。患者，女，67岁。结节最大径18.9 mm，实性成分最大径9.8 mm，CTR 51.85%，纵隔窗最大径3.8 mm，TDR 20.11%。术后病理为IAC。 CT of patients with IAC. The patient was a 67-year-old woman. The diameter of the nodule was 18.9 mm, the diameter of the solid component was 9.8 mm, CTR was 51.85%, the diameter of mediastinal window nodule was 3.8 mm. TDR was 20.11%. The postoperative pathology was IAC. IAC: invasive adenocarcinoma.

三维图像处理：重建薄层CT图像，并将其导入Synapse 3D软件(日本东京富士医疗公司)。两名胸部放射科医生和一名胸部外科医生独立观察这些3D图像，标记出感兴趣区域后由软件自动计算出肺结节的总体积、实性成分体积以及实性成分体积占比、肺结节平均CT值，在分析前对数据进行了匿名处理，标记感兴趣区域时注意剔除肺血管对实性成分的干扰。

### 1.3 统计分析

采用SPSS 26.0统计软件，符合正态分布的计量资料以均数±标准差(Mean±SD)表示，使用独立样本*t*检验; 不符合正态分布的计量资料以M(Q1, Q3)表示，采用*Mann-Whitney U*检验; 计数资料采用χ^2^检验; 同时将*P* < 0.05的定量资料分别按二维与三维分类纳入进行多因素*Logistic*回归分析，将独立危险因素的计量资料绘制为ROC曲线，计算曲线下面积(area under the curve, AUC)，并求得cut-off值，以*P* < 0.05为差异有统计学意义。

## 2 结果

### 2.1 逻辑回归分析结果

IAC组与非IAC组(MIA和PGL)比较，除性别外，差异均有统计学意义(表 1)，将有统计学差异的定量资料分别按二维与三维分类纳入多因素*Logistic*回归分析，发现二维因素组多元逻辑回归中：年龄(OR=1.024, 95%CI：1.006-1.043, *P*=0.009)、结节最大径(OR=1.195, 95%CI: 1.137-1.256, *P* < 0.001)、实性成分最大径(OR=1.196, 95%CI: 1.102-1.298, *P* < 0.001)、纵隔窗结节最大径(OR=1.167, 95%CI: 1.044-1.304, *P*=0.007)是预测结节IAC的独立危险因素(表 2)。三维因素多元逻辑回归中，由于结节总体积与结节实性成分体积组内变异大，以cm^3^为单位纳入回归，年龄(OR=1.035, 95%CI: 1.018-1.052, *P* < 0.001)、结节总体积(OR=1.855, 95%CI: 1.346-2.556, *P* < 0.001)、实性部分体积(OR=1.458, 95%CI: 1.270-1.674, *P* < 0.001)、平均CT值(OR=1.439, 95%CI: 1.337-1.550, *P* < 0.001)均是预测结节IAC的独立危险因素(表 3)。

**表 1:** IAC与非IAC组患者各因素对比 Comparison of factors between IAC and non IAC patients

Factors		Pathological type		*χ*^2^/*t*/*U*	*P*
		Non-IAC (*n*=316)	IAC (*n*=470)		
Gender	Male	31 (9.8%)	47 (10.0%)	0.008	0.930
	Female	285 (90.2%)	423 (90.0%)		
Age (yr)		50.31±11.36	58.40±10.62	10.179	< 0.001
Nodule volume (mm^3^)		346.60 (207.33, 618.08)	1, 298.15 (661.10, 2, 737.40)	25, 791.500	< 0.001
Solid partial volume (mm^3^)		9.45 (0.23, 40.33)	257.60 (66.28, 778.58)	20, 336.000	< 0.001
Proportion of solid component volume (%)		2.50 (0.10, 9.75)	21.45 (8.28, 46.75)	29, 677.500	< 0.001
Average CT value (Hu)		-613.05 (-676.10, -536.35)	-485.35 (-591.23, -331.15)	39, 520.000	< 0.001
Nodule diameter (mm)		9.95 (8.30, 12.20)	16.85 (12.20, 21.80)	25, 744.500	< 0.001
Solid component diameter (mm)		1.65 (0.00, 4.10)	8.13 (4.00, 14.20)	25, 846.500	< 0.001
Mediastinal window nodule diameter (mm)		0.00 (0.00, 0.00)	4.80 (0.00, 10.53)	32, 797.000	< 0.001
CTR (%)		17.34 (0.00, 39.62)	52.67 (28.07, 79.83)	35, 848.000	< 0.001
TDR (%)		100.00 (100.00, 100.00)	69.44 (39.57, 100.00)	35, 448.500	< 0.001

**表 2:** CT二维特征的多因素逻辑回归结果 Multivariate *Logistic* regression analysis of CT two-dimensional factors

Factors	Wald	*P*	OR	95%CI
Year	6.872	0.009	1.024	1.006-1.043
Nodule diameter	49.151	< 0.001	1.195	1.137-1.256
Solid component diameter	18.340	< 0.001	1.196	1.102-1.298
Mediastinal window nodule diameter	7.361	0.007	1.167	1.044-1.304

**表 3:** CT三维特征的多因素逻辑回归结果 Multivariate *Logistic* regression analysis of CT three-dimensional factors

Factors	Wald	*P*	OR	95%CI
Year	16.175	< 0.001	1.035	1.018-1.052
Nodule volume	36.493	< 0.001	1.855	1.346-2.556
Solid partial volume	31.282	< 0.001	1.458	1.270-1.674
Average CT value	93.113	< 0.001	1.439	1.337-1.550

### 2.2 ROC曲线分析结果

将年龄、结节最大径、实性成分最大径、纵隔窗结节最大径、CTR、结节总体积、实性部分体积、平均CT值、实性成分体积占比、1-TDR进行ROC曲线分析。IAC与非IAC二维层面鉴别的年龄cut-off值约为55.50(敏感度为64.7%，特异度为67.7%)，结节最大径cut-off值为12.55 mm(敏感度为74.0%，特异度为77.8%)，实性成分最大径cut-off值为5.65 mm(敏感度为62.6%，特异度为89.9%)，纵隔窗最大径cut-off值为2.05 mm(敏感度为64.3%，特异度为84.5%)，CTR的cut-off值为25.77%(敏感度为78.3%，特异度为61.1%)，1-TDR(即纵隔窗结节最大径/肺窗结节最大径)的cut-off值为6.71%(敏感度为67.7%，特异度为79.1%); 三维层面结节总体积cut-off值为681.25 mm^3^(敏感度为74.5%，特异度为79.1%)，实性部分体积cut-off值为49.10 mm^3^(敏感度为80.4%，特异度为79.7%)，实性成分体积占比cut-off值为9.05%(敏感度为73.4%，特异度为73.7%)，平均CT值cut-off值为-515.95 Hu(敏感度为58.3%，特异度为79.7%)(表 4)。通过约登指数(敏感度+特异度-1)将各项指标诊断效能由大到小排序：实性部分体积(0.601) > 结节总体积(0.536) > 实性成分最大径(0.525) > 结节最大径(0.518) > 纵隔窗结节最大径(0.488) > 实性成分体积占比(0.471) > 1-TDR(0.468) > CTR(0.394) > 平均CT值(0.380) > 年龄(0.324)。二维与三维对应参数对比时，三维均优于二维(结节总体积优于结节最大径，实性成分体积优于实性成分最大径，CTR优于实性成分体积占比)(图 3)。

**表 4:** 各计量资料ROC曲线结果 ROC curve factors

Factors	Cut-off value	AUC	Sensitivity (%)	Specificity (%)	Youden index
Year	55.50	0.702	64.7	67.7	0.324
Nodule volume	681.25	0.826	74.5	79.1	0.536
Solid partial volume	49.10	0.863	80.4	79.7	0.601
proportion of solid component volume (%)	9.05	0.800	73.4	73.7	0.471
Average CT value	-515.95	0.734	58.3	79.7	0.380
Nodule diameter	12.55	0.827	74.0	77.8	0.518
Solid component diameter	5.65	0.826	62.6	89.9	0.525
Mediastinal window nodule diameter	2.05	0.779	64.3	84.5	0.488
CTR (%)	25.77	0.759	78.3	61.1	0.394
1-TDR (%)	6.71	0.761	67.7	79.1	0.468
AUC: area under the curve; ROC: receiver operating characteristic.					

**图 3:**
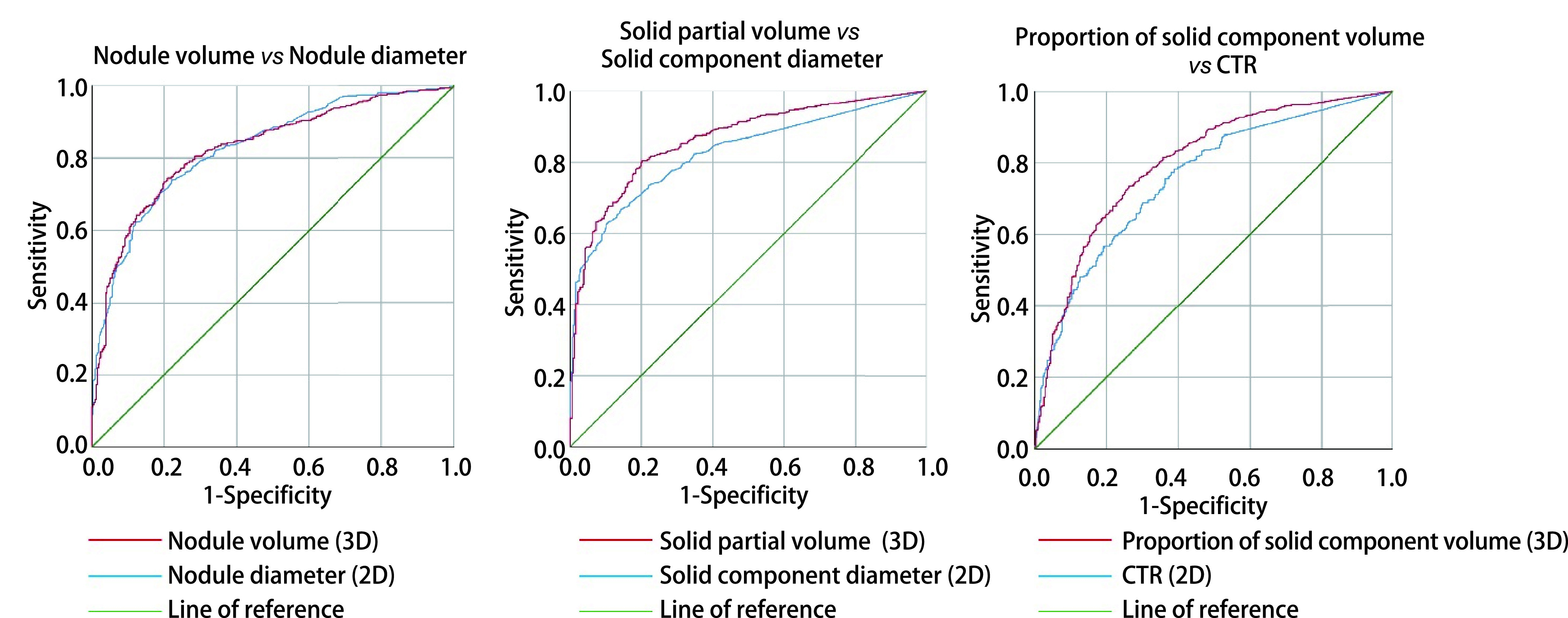
二维与三维因素对比 Comparison of two-dimensional and three-dimensional factors

## 3 讨论

现有研究^[16]^表明，AAH、AIS、MIA三种病理类型预后良好，而IAC患者则相对预后较差，将IAC与其他三种病理类型的肺腺癌区分对患者手术方式的选择以及预后的判断具有一定的临床意义。已有很多研究对胸部CT的二维特征进行分析，随着读片软件的进步，也出现了一些对CT的三维特征进行比较的研究，但对比二维与三维特征对IAC诊断能力的研究仍然较少。本文从二维层面的测量指标如肺窗结节最大径、结节实性成分最大径、纵隔窗结节最大径、CTR、TDR等特征以及三维层面的肺结节的总体积、实性成分体积以及实性成分体积占比、肺结节平均CT值以及患者的基线特征几个方面对比了二维及三维特征对IAC的诊断价值。其中，针对结节三维特征进行测量的实性部分体积与结节总体积拥有最佳的IAC诊断能力，测量结节二维特征的实性成分最大径与结节最大径次之，计算实性成分相对结节整体比例的指标实性成分体积占比以及CTR稍差。在结节整体特征、结节实性成分特征、CTR三类指标中，三维层面均优于二维层面。实性成分体积具有最大的敏感度(81.6%)，实性成分最大径特异度最大(89.7%)，实性成分的大小较结节总体大小更能准确预测肺结节性质。虽然目前CTR被广泛用于作为评估肺结节良恶性以及选择手术方式的指标^[17]^，但在我们的研究中，实性成分体积占比较CTR有更优的检验能力。纵隔窗最大径与TDR以及平均CT值也可作为鉴别IAC的标准。

对于本文得出三维指标优于二维指标的结论，我们认为可能是由于以下原因：①三维的指标相较于二维指标更能反映结节整体特征，目前测量的大部分二维指标均在水平面上获得，而结节向各个方向生长，并不会局限于某一个特定方向或层面; ②三维指标更不易受到结节形状或实性成分分布的影响，例如当结节实性成分分散时，二维测量实性成分最大径会出现很大的误差，测算三维层面的结节实性体积则不会受此影响。

本研究分析的数据中，女性患者(*n*=708)显著多于男性患者(*n*=78)，这可能预示着女性患者肺腺癌发病率显著高于男性患者，但就诊断IAC而言，性别并无统计学差异。年龄是诊断IAC的独立危险因素，55.5岁以上的患者更倾向于诊断IAC。

目前有研究^[18]^得出结论：病灶直径≥1.39 cm、平均CT值-597.45 Hu作为鉴别IAC与MIA的分割点，这与本研究结果相近; 丁宁等^[19]^的研究纳入了120例患者，结节直径≥19.22 mm、实性成分直径≥5.23 mm作为诊断IAC的分割点，与本研究结果有差异，可能是由于其纳入病例均为混合磨玻璃结节，而本文并未排除纯磨玻璃结节; 顾鑫蕾等^[20]^的研究中，肺窗最大径≥14.5 mm、实性成分比例≥5%时，诊断IAC的可能性更大，其中结节最大径的结论与我们的研究结果相近，CTR与我们的结论相差较大，这可能是由于作者纳入研究纯磨玻璃结节比例较大; 在Ding等^[21]^的研究中，混合磨玻璃区分AIS及MIA组与IAC组的结节直径cut-off值为15.4 mm，实性成分直径cut-off值为5.8 mm，皆与本文结论相近; Suzuki等^[10]^将结节最大径≥2 cm、CTR≥0.25定义为放射学IAC，其中CTR值与我们的研究接近，但其仅针对0.25、0.5、0.75三个截断点对比了其检验效能，并未进行进一步统计学分析寻找最佳截断点。

代平等^[22]^的研究中，结节体积无统计学意义，这可能是由于作者纳入IAC患者数量较少(*n*=36)，样本量不足导致数据分布存在较大误差。Saeki等^[23]^的研究中，实性成分体积诊断IAC的cut-off值为75 mm^3^，与我们的49.10 mm^3^差距较大，可能是由于作者分析了直径≤2 cm的结节，而本文纳入了直径≤3 cm的结节，直径为2 cm-3 cm的结节即使实性成分较低，浸润程度也会上升。

我们的研究通过缜密的统计学方法证实了胸部CT的三维特征检验肺结节浸润性效能高于二维特征。临床医生更多依靠肺结节三维特征预测肺腺癌浸润性是一种新的趋势。

本文尚存在一定局限性，首先我们只分析了单个中心的数据，并不能代表其他中心情况，可能与其他中心结论有所差异。其次由于手术患者的选择具有一定倾向性，CT特征倾向于早期病变的部分患者尚处于随访中，且本文删除了直径 > 3 cm的肺腺癌患者，这可能导致了数据偏倚，且PGL患者数量较少。对比二维与三维CT特征时没有匹配同质特征，仅统计了常见的几种CT特征，可以在此基础上补充二维层面的结节面积、实性成分面积; 三维上的最大径^[4]^、实性成分最大径、纵隔窗肺结节最大径等，这有待于进一步的研究完善。此外，已有研究^[14, 24, 25]^表明通过组合两种或以上的CT特征可以得到更优的诊断结果，将各项现有的CT特征结合，找出更优的综合评估肺结节浸润程度的指标也是一种新的研究方向。
